# Giant cell tumor of the uterus: case report and response to chemotherapy

**DOI:** 10.1186/1471-2407-7-46

**Published:** 2007-03-14

**Authors:** Keith M Skubitz, J Carlos Manivel

**Affiliations:** 1 Department of Medicine, University of Minnesota Medical School, and the Masonic Cancer Center, Minneapolis, MN 55455, USA; 2 Department of Laboratory Medicine and Pathology, University of Minnesota Medical School, and the Masonic Cancer Center, Minneapolis, MN 55455, USA

## Abstract

**Background:**

Giant cell tumor (GCT) is usually a benign but locally aggressive primary bone neoplasm in which monocytic macrophage/osteoclast precursor cells and multinucleated osteoclast-like giant cells infiltrate the tumor. The etiology of GCT is unknown, however the tumor cells of GCT have been reported to produce chemoattractants that can attract osteoclasts and osteoclast precursors. Rarely, GCT can originate at extraosseous sites. More rarely, GCT may exhibit a much more aggressive phenotype. The role of chemotherapy in metastatic GCT is not well defined.

**Case presentation:**

We report a case of an aggressive GCT of the uterus with rapidly growing lung metastases, and its response to chemotherapy with pegylated-liposomal doxorubicin, ifosfamide, and bevacizumab, along with a review of the literature.

**Conclusion:**

Aggressive metastasizing GCT may arise in the uterus, and may respond to combination chemotherapy.

## Background

Giant cell tumor (GCT) of bone, also known as osteoclastoma, is a primary osteolytic bone neoplasm in which monocytic macrophage/osteoclast precursor cells and multinucleated osteoclast-like giant cells infiltrate the tumor [1-3]. Walker first demonstrated that osteoclasts are derived from monocytic osteoclast precursors in blood [4,5]. The origin of GCT is unknown, but the tumor cells of GCT have been reported to produce chemoattractants that can attract osteoclasts and their precursors [3,6].

GCT is usually benign but locally aggressive, and most commonly occurs in the epiphysis of long bones. Rarely, GCT can originate at extraosseous sites. Metastases from GCT of bone are unusual, and often behave in an indolent manner that can be managed by surgery [7]. More rarely, GCT may exhibit a much more aggressive phenotype. The role of chemotherapy in metastatic GCT is not well defined. We report a case of an aggressive GCT of the uterus with rapidly growing lung metastases, and its response to chemotherapy with pegylated-liposomal doxorubicin, ifosfamide, and bevacizumab, along with a review of the literature.

## Case presentation

**Figure 1:**
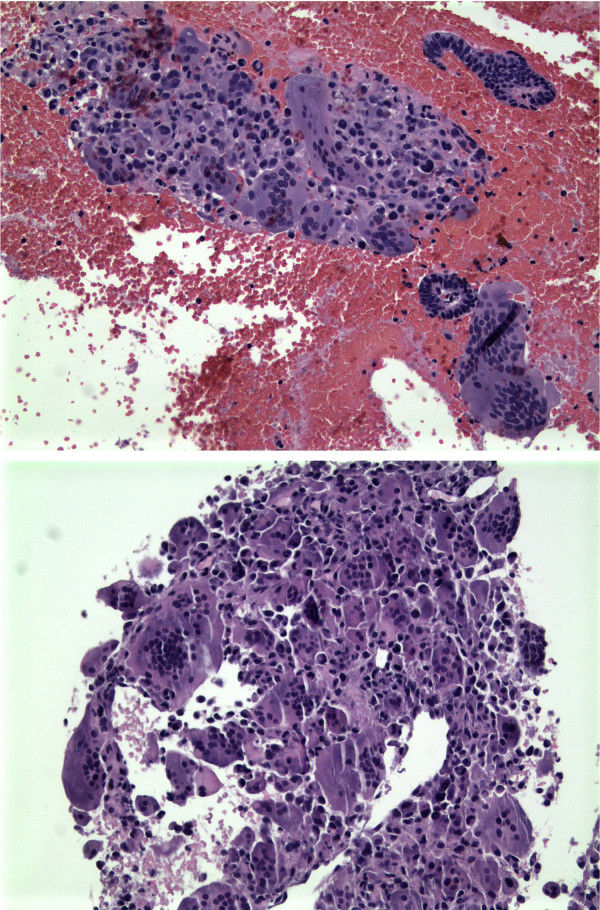
Top, endometrial curetting showed aggregates of plump mononuclear cells with numerous large multinucleated osteoclast-type giant cells. Benign endometrial glands are seen at right center and right upper corner. Bottom, Fine needle aspirate of the lung showed sheets of mononuclear plump cells and numerous scattered osteoclast-type giant cells. See the text for complete pathologic description.

A 55 year old woman developed anorexia, vaginal bleeding, and a non-productive cough over one month. Her last menstrual period was 11 months earlier, and she had noted some vaginal spotting for 5 months. CT scans revealed a large uterine mass and multiple lung nodules. An endometrial curetting showed small fragments of tumor consisting of two components; aggregates of atypical plump mononuclear cells with hyperchromatic, pleomorphic nuclei were interspersed with numerous large multinucleated osteoclast-type giant cells, some of which had more than 30 benign-appearing nuclei (Figure 1, Top). Up to ten mitoses power high-power field were observed in the mononuclear cells. Immunoperoxidase stain for the histiocytic marker CD68 was positive in a subpopulation of mononuclear cells and in the osteoclast-type giant cells. These cells were negative for the epithelial marker cytokeratin (AE1/AE3) which highlighted scattered benign endometrial glands. A fine needle aspirate of the lung showed sheets of atypical mononuclear plump cells and numerous scattered benign-appearing osteoclast-type giant cells (Figure 1, Bottom). Some mononuclear cells were positive for the endometrial stromal marker CD10; all mononuclear and multinucleated cells were positive for the mesenchymal cell marker vimentin; CD117 (c-kit) was weakly positive in multinucleated cells. All cells were negative for estrogen receptors, cytokeratins (7, 20, AE1/AE3), muscle specific actin and mucin.

**Figure 2:**
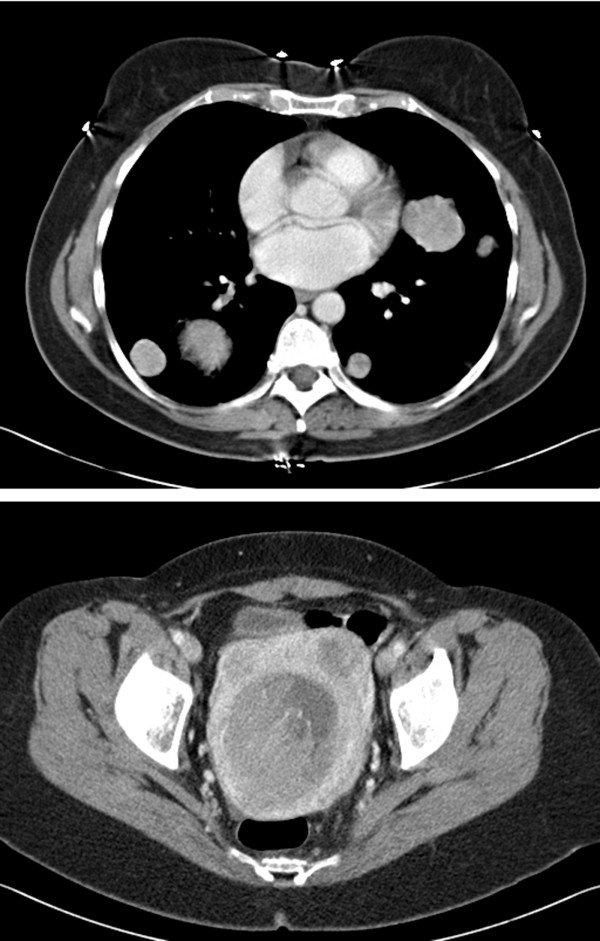
CT scan at start of chemotherapy.

**Figure 3:**
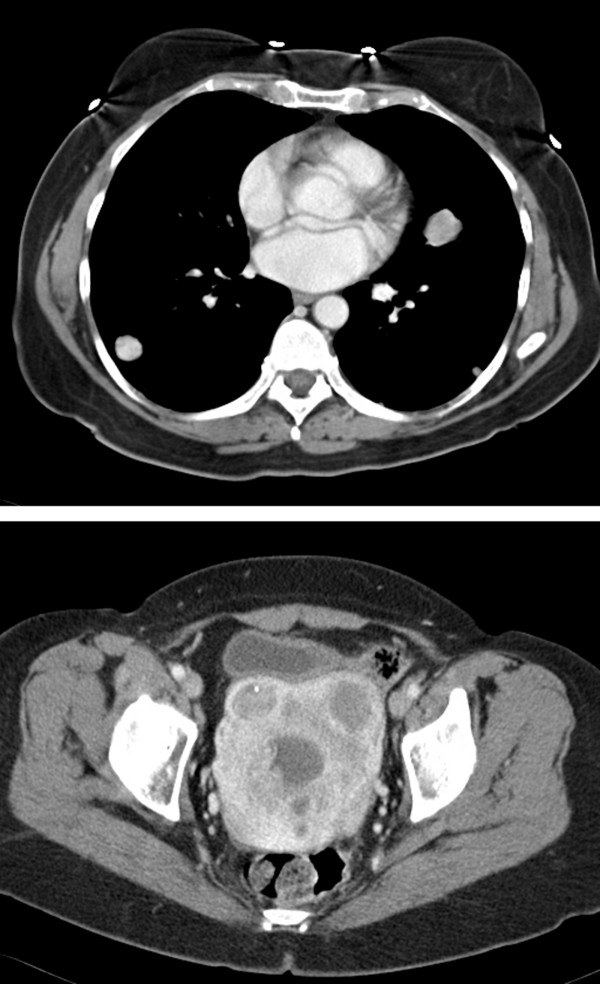
CT scan at start of chemotherapy cycle #2.

**Figure 4:**
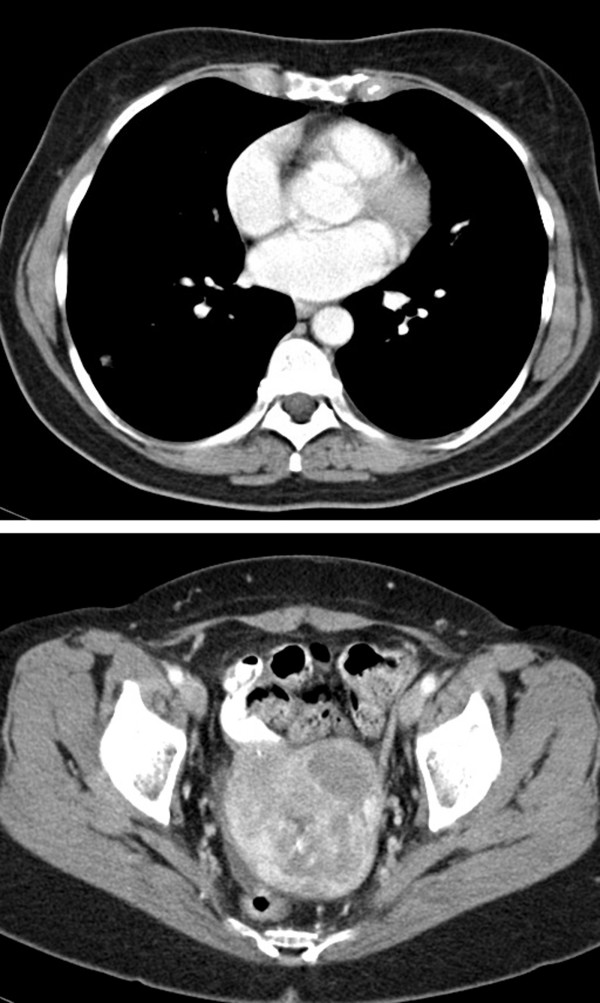
CT scan at start of chemotherapy cycle #8.

On presenting to our clinic, her history was remarkable for anorexia, uterine bleeding, dyspnea on exertion, a non-productive cough, and a 10 pound weight loss during the previous month. Her past history was notable for treated hypertension and hypothyroidism, an episode of gout, and monoclonal gammopathy of undetermined significance. Her medications were lisinopril, L-thyroxine, and a multivitamin. Her physical examination was notable for scattered crackles and occasional wheezes, and mild lower abdominal tenderness and fullness. A repeat CT scan revealed prominent tumor progression during the preceeding month, with a 11.6 cm uterine mass and lung nodules, some >4 cm in diameter (Figure 2). Hemoglobin was 8.5 g/dl, AST, alkaline phosphatase, bilirubin, creatinine, and electrolytes were normal. Treatment was begun with pegylated-liposomal doxorubicin (45 mg/m2, day 1), ifosfamide (9 g/m2 total dose given by continuous infusion over 6 days, days 1–6) with mesna uroprotection [8], and bevacizumab 5 mg/kg every 2 weeks, with pegfilgrastim 6 mg given subcutaneously on day 7, with cycles repeated every 28 days. After the first cycle of chemotherapy, repeat CT imaging revealed a tumor response in the lung nodules (Figure 3), although her symptoms were unchanged. At the start of cycle 3 her cough, vaginal bleeding, and dyspnea on exertion had noticeably improved. The dose of pegylated-liposomal doxorubicin was decreased by 10% for the third and subsequent cycles due to mucositis and skin rash. A CT scan before cycle 4 showed continued tumor regression. Blood pressure remained controlled and no proteinuria was noted. After 7 cycles the tumor had stabilized by CT imaging (Figure 4). The lung nodules had largely disappeared, but the uterine mass remained at about 8.5 cm. She was given cycle 8 without bevacizumab in anticipation of hysterectomy. Repeat CT imaging showed stable disease, and she underwent a total abdominal hysterectomy with bilateral salpingo-oophorectomy 8 weeks after cycle 8.

**Figure 5:**
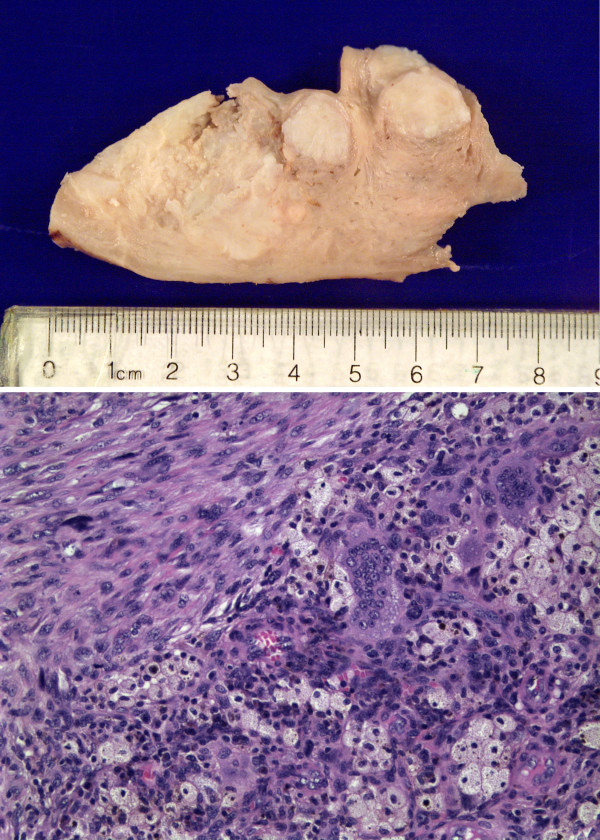
Top, the hysterectomy specimen contained residual poorly demarcated tumor (left) and multiple leiomyomata (right). Bottom, sections from the uterine tumor showed residual mononucleated plump to spindle shaped tumor cells (left upper corner) and inflammatory cells, including foamy macrophages, osteoclast-type giant cells, and histiocytes (lower right). See the text for complete pathologic description.

The uterus contained a poorly demarcated tumor that measured 4.8 × 4.0 × 3.5 cm arising in the left lateral wall (Figure 5, Top). Multiple leiomyomata ranging from 0.3 to 2.2 cm were also identified. Sections from the tumor (Figure 5, Bottom) showed atypical mononucleated plump to spindle shaped tumor cells diffusely infiltrating the myometrium and within dilated blood vessels. Tumor cells accounted for approximately 30 % of the cells. Vascular invasion was observed. Occasional multinucleated giant cells of osteoclastic-type were also recognized; however, these were rare and tended to form small aggregates. Extensive areas of necrosis showed dystrophic calcification. Large numbers of histiocytes with foamy cytoplasm and hemosiderin granules were observed throughout the tumor; these cells accounted for approximately 70% of the cellular population. A subpopulation of mononuclear cells was positive for CD10; osteoclastic cells were non-reactive. Some of the mononuclear plump and spindle cells were weakly positive for actin; they were negative for desmin and smooth muscle myosin. CD117 was weakly positive in the osteoclastic cells. The cell division marker Ki67 was positive in tumor cells and negative in histiocytes and osteoclasts; Stains for common leukocyte antigen (CD45) and for the histiocytic marker CD68 were positive in histiocytes and osteoclasts, but were negative in tumor cells. Stains for lysozyme were weakly positive in histiocytes and osteoclasts but were negative in tumor cells. Stains for cytokeratin (AE1/AE3), estrogen receptors, and progesterone receptors were negative. Pelvic washings were negative; the cervix, parametria, tubes, and ovaries were free of tumor.

Two months after the hysterectomy, progression of the lung nodules was noted on CT imaging and therapy with pegylated-liposomal doxorubicin and bevacizumab was begun, but progression was noted after 2 months. Gemcitabine (675 mg/m2 intravenously over 90 min given days 1 and 14 with pegfilgrastim days 2 and 15 due to neutropenia on day 8 of the first cycle, with cycles repeated every 28 days) [9,10] with bevacizumab was begun, but progression was noted after two months. Therapy was then begun with ifosfamide and etoposide [8] and bevacizumab, and a partial response was observed after two months, with stabilization at 4 months. The patient received a total of 6 cycles of ifosfamide, etoposide, bevacizumab, although bevacizumab was held on day 14 of cycle 6 due to proteinuria. Two months later significant progression was noted. She received 1 cycle of dacarbazine, mitomycin c, and cisplatinum, with a very good response, but prominent toxicity including myelosuppression and fatigue. Two months later tumor progression was noted.

## Conclusion

The current report describes an unusual case of aggressive GCT of the uterus and its response to chemotherapy with pegylated-liposomal doxorubicin, ifosfamide, and bevacizumab. The role of chemotherapy in GCT is not well defined. Pegylated-liposomal doxorubicin and ifosfamide are active in soft tissue and bone sarcomas [8,11-15]. Angiogenic growth factor expression, including VEGF, has been previously reported in a variety of sarcomas and in GCT of bone [16-23], providing a rationale for the potential role of therapy directed against the VEGF signaling pathway, such as bevacizumab, in sarcomas, including GCT. In addition, VEGF is capable of inducing osteoclastogenesis in osteopetrotic mice (op/op) [24]. The current case responded well to therapy with pegylated-liposomal doxorubicin, ifosfamide, and bevacizumab. It is of interest that in the first two specimens obtained before treatment (endometrial curetting and lung aspirate) there were large numbers of giant cells and these were evenly distributed. In contrast, the hysterectomy specimen after chemotherapy, showed only rare osteoclast-type giant cells, and there was extensive replacement of tumor cells by necrosis and reactive histiocytes.

Usually GCT is a primary osteolytic bone neoplasm in which monocytic macrophage/osteoclast precursor cells and multinucleated osteoclast-like giant cells infiltrate the tumor [1-3,6]. These multinucleated giant cells have biochemical and functional characteristics of osteoclasts (reviewed in [18]). While usually benign, GCT is locally aggressive and most commonly occurs in the epiphysis of long bones. GCT of soft parts are very rare, but have been described [25,26], as have GCT of the thyroid [27] and pancreas [28,29].

Pulmonary metastases of GCT of bone usually grow slowly, and are often treated by surgery, though in some cases chemotherapy with various agents or radiation therapy have been used [30-39]. Two cases of rapidly progressive metastatic GCT of bone have been reported that were treated with chemotherapy with a transient response [40,41]. Several reports have suggested the potential utility of interferon alpha in GCT of bone [30,42-44].

Walker observed that osteoclasts are derived from monocytic progenitors found in the blood [4,5], providing the first demonstration of the existence of stem cells in blood for non-hematopoietic tissue. The etiology of GCT is unknown, however the tumor cells of GCT have been reported to produce chemoattractants that can recruit osteoclasts and osteoclast precursors [3,6].

Many reactive conditions and benign and malignant tumors contain osteoclast-type giant cells. An important difference between many of these entities and the "true" giant cell tumor is that the spatial relationship between the giant cells and the tumor cells is different. In giant cell tumor, the osteoclast-type giant cells are distributed regularly and uniformly throughout the tumor; in other entities, the giant cells tend to be clustered, frequently in areas of hemorrhage, that alternate with areas completely devoid of giant cells. This difference in distribution probably reflects differences in pathogenesis. It has been suggested that in the various entities with unevenly distributed giant cells, the latter are probably the result of cytokines produced that attract osteoclast precursors. In giant cell tumor, the even distribution may reflect interaction/co-dependence as in a paracrine loop between the tumor stromal cells and the reactive, osteoclast type giant cells.

We found reports of 9 other cases of GCT of the uterus [45-51]. Three patients in whom follow up was available died of their tumors. Four of the cases were thought to also have smooth muscle differentiation. Absence of desmin and myosin and only focal and weak reactivity for actin argue against true smooth muscle differentiation in our case.

The tumor cells of GCT of bone have been reported to produce chemoattractants, including both TGF beta1 and monocyte chemoattractant protein 1 (MCP-1), that can attract osteoclasts and TRAP-positive monocytic osteoclast precursors. Other growth factors indicative of an osteoclastogenic environment of potential importance in GCT, including RANKL, have been identified in GCT [6,18,52-54]. RANKL (OPGL) binds specific hematopoietic progenitor cells and induces osteoclast differentiation. RANKL also activates osteoclasts, and administration of RANKL to mice results in hypercalcemia [55]. RANKL is necessary and sufficient for osteoclastogenesis [52]. RANKL mRNA expression was recently reported to be over-expressed in the stromal-like tumor cells of GCT, while its receptor, RANK, was expressed only in the macrophage-like mononuclear cells and multinucleated giant cells [6,52-54]. The potential role for tumor cell-osteoclast interaction/co-dependence, as in a paracrine loop between the stromal tumor cells and the osteoclast-like cells, in GCT is unknown. In this regard, it is of interest that the marked decrease in the population of viable tumor cells in the hysterectomy specimen after chemotherapy was associated with a marked reduction in the number of osteoclast-type giant cells and with loss of their spatial relationship. Thus, it is intriguing to speculate that new agents that inhibit osteoclastogenesis via the RANK/RANKL pathway, such as denosumab, could be useful in the treatment of GCT. Recent studies also suggest that bisphosphonates may induce apoptosis in both osteoclast-like giant cells and stromal tumor cells in vivo and in vitro in GCT [56,57], possibly by interfering with an autocrine and/or paracrine loop in the tumor between stromal tumor cells and osteoclast-like giant cells.

Aggressive metastasizing GCT may arise in the uterus, and may respond to combination chemotherapy. The addition of bevacizumab is logical, though its contribution to the effect observed in this case is unclear.

## Abbreviations

GCT, giant cell tumor; VEGF, vascular endothelial growth factor; TGF, transforming growth factor; TRAP, tartrate-resistant acid phosphatase; RANKL, receptor activator of NF-kappa B ligand; OPGL, osteoprotegrin ligand.

## Competing interests

KMS owns publicly traded stock in Genentech, and Johnson & Johnson, is on speakers bureaus for Johnson & Johnson, is taking part in a trial using a drug made by Amgen (denosumab) for the treatment of GCT, and has taken part in trials with drugs made or marketed by Amgen, Johnson & Johnson, Bristol Myers, and Genentech.

## Authors' contributions

KMS participated in care of the patient, data analysis, and helped draft the manuscript.

JCM helped analyze data, performed the pathologic examination, and helped draft the manuscript. Both authors read and approved the final manuscript.

## Pre-publication history

The pre-publication history for this paper can be accessed here:


